# MRI Texture Analysis Reveals Brain Abnormalities in Medically Refractory Trigeminal Neuralgia

**DOI:** 10.3389/fneur.2021.626504

**Published:** 2021-02-12

**Authors:** Hayden Danyluk, Abdullah Ishaque, Daniel Ta, Yee Hong Yang, B. Matthew Wheatley, Sanjay Kalra, Tejas Sankar

**Affiliations:** ^1^Division of Surgical Research, Department of Surgery, University of Alberta, Edmonton, AB, Canada; ^2^Division of Neurosurgery, Department of Surgery, University of Alberta Hospital, University of Alberta, Edmonton, AB, Canada; ^3^Neuroscience and Mental Health Institute, University of Alberta, Edmonton, AB, Canada; ^4^Department of Computing Science, University of Alberta, Edmonton, AB, Canada

**Keywords:** trigeminal neuralgia, chronic pain, neuroimaging, magnetic resonance imaging, texture analysis, anterior cingulate, insula, thalamus

## Abstract

**Background:** Several neuroimaging studies report structural alterations of the trigeminal nerve in trigeminal neuralgia (TN). Less attention has been paid to structural brain changes occurring in TN, even though such changes can influence the development and response to treatment of other headache and chronic pain conditions. The purpose of this study was to apply a novel neuroimaging technique—texture analysis—to identify structural brain differences between classical TN patients and healthy subjects.

**Methods:** We prospectively recruited 14 medically refractory classical TN patients and 20 healthy subjects. 3-Tesla T1-weighted brain MRI scans were acquired in all participants. Three texture features (autocorrelation, contrast, energy) were calculated within four *a priori* brain regions of interest (anterior cingulate, insula, thalamus, brainstem). Voxel-wise analysis was used to identify clusters of texture difference between TN patients and healthy subjects within regions of interest (*p* < 0.001, cluster size >20 voxels). Median raw texture values within clusters were also compared between groups, and further used to differentiate TN patients from healthy subjects (receiver-operator characteristic curve analysis). Median raw texture values were correlated with pain severity (visual analog scale, 1–100) and illness duration.

**Results:** Several clusters of texture difference were observed between TN patients and healthy subjects: right-sided TN patients showed reduced autocorrelation in the left brainstem, increased contrast in the left brainstem and right anterior insula, and reduced energy in right and left anterior cingulate, right midbrain, and left brainstem. Within-cluster median raw texture values also differed between TN patients and healthy subjects: TN patients could be segregated from healthy subjects using brainstem autocorrelation (*p* = 0.0040, AUC = 0.84, sensitivity = 89%, specificity = 70%), anterior insula contrast (*p* = 0.0002, AUC = 0.92, sensitivity = 78%, specificity = 100%), and anterior cingulate energy (*p* = 0.0004, AUC = 0.92, sensitivity = 78%, specificity = 100%). Additionally, anterior insula contrast and duration of TN were inversely correlated (*p* = 0.030, Spearman r = −0.73).

**Conclusions:** Texture analysis reveals distinct brain abnormalities in TN, which relate to clinical features such as duration of illness. These findings further implicate structural brain changes in the development and maintenance of TN.

## Introduction

Trigeminal neuralgia (TN) is a chronic, neuropathic facial pain disorder characterized by intermittent, typically unilateral, electric shock-like or stabbing pain attacks in the distribution of one or more branches of the trigeminal nerve (cranial nerve V—CNV) (1). The condition is severely disabling, often fails to respond long-term to medications against neuropathic pain, and has historically been associated with a high suicide rate (2). While a subset of TN is associated with brain lesions (e.g., demyelinating plaques in multiple sclerosis, or tumor), the most common form is “classical” TN, in which neurovascular compression of CNV, typically at its root entry zone, is observed (1). In these patients, microvascular decompression (MVD) can be an effective surgical treatment, though rates of post-operative pain recurrence may be as high as 25% at 2 years, and 4% per year thereafter (3).

Considerable attention has been paid to the structure of CNV in classical TN, which has been studied primarily using magnetic resonance imaging (MRI) diffusion tensor imaging (DTI) (4–7). However, a nerve-centric conceptualization inadequately explains all features of TN, including, among others, the development of medication-refractoriness, variability in response to treatment, and other consistent observations such as the greater frequency of right-sided compared to left-sided TN (3, 8–10). Recent investigations have identified abnormalities at more proximal locations along the trigeminal pathway in classical TN, more specifically within the brainstem (11–13). Additionally, structural abnormalities in several different brain regions have also been observed, including in the anterior cingulate cortex (ACC), insula, thalamus, and hippocampus (14–17), the latter which we have previously shown to be related to the durability of pain relief after surgical treatment (17).

Texture analysis is a statistical method by which gray-level intensity and patterns that cannot be detected by the human eye are quantified in images (including MRI scans) at an individual subject level, and then compared between groups of interest (18, 19). Developed decades ago for the assessment of aerial photographs (20), this technique has been applied to neuroimaging analyses across various neurological conditions including Alzheimer's disease (21), amyotrophic lateral sclerosis (22, 23), temporal lobe epilepsy (24), and multiple sclerosis (25). To date, texture analysis has neither been applied to the study of TN, nor—to our knowledge—to any other headache or chronic pain condition except for a single prior investigation of textural abnormalities in medication-overuse headache (26).

Our central hypothesis was that texture analysis can identify subtle structural brain abnormalities in MRI scans of patients with classical TN, and further that these abnormalities may relate to clinical features including pain severity and duration of illness. Additionally, given differences in the incidence of right- vs. left-sided TN, we also predicted that texture features might show different hemispheric lateralization between left- and right-TN patients. Our primary objective was therefore to perform a focused search for texture differences between classical TN patients and healthy control subjects. Given the known association between multiple sclerosis and TN, we examined texture features—*contrast* and *energy*—previously shown to be abnormal in patients with multiple sclerosis or clinically isolated syndrome, under the assumption that these features might uncover potential subtle structural lesions unapparent to the human eye (25). We also evaluated *autocorrelation* because it has been shown to correlate with changes in brain tissue diffusion (22). Our analysis was restricted to four *a priori* brain regions known to be structurally abnormal in other chronic pain conditions (ACC, insula, thalamus, and brainstem) (14–17), and was further divided into three sub-analyses designed to evaluate left-right and ipsilateral-contralateral structural differences in TN.

## Materials and Methods

### Subjects

Fourteen patients with classical TN were prospectively recruited for this study. Every patient had at least a partial initial response to carbamazepine or oxcarbazepine, but all were considered medically-refractory at the time of enrolment based on two criteria: (1) their TN pain was no longer adequately managed by any combination of TN medications; or (2) they were unable to either escalate medication dose or try new medications (due to side effects or unwillingness to consent to new medications). Consequently, these patients provided informed consent for MVD surgery, and were awaiting surgery at the time of recruitment. No patients had previously undergone surgical treatment for TN. All patients were diagnosed with classical TN according to the criteria of the International Classification of Headache Disorders, 3rd edition (1). The presence of neurovascular compression was demonstrated in all patients on routine pre-operative imaging with MRI, using standard high-resolution T2-weighted sequences. Patients presenting with bilateral TN or a potential lesional cause of TN were excluded, as well as patients with other underlying chronic pain, neurological or psychiatric disorders. All patient imaging and pain assessments were performed within 1 month of surgical treatment. We also included 20 healthy control subjects without history of any neurological, psychiatric, or chronic pain disorders. The study was approved by the Human Research Ethics Board at the University of Alberta, and all participants provided written informed consent prior to enrolment.

### MRI Acquisition

All imaging data was collected with a Siemens Prisma Magnetom 3 Tesla MRI scanner (Siemens Medical System, Erlangen, Germany) using a 64-channel head-coil. For texture analysis, 3D magnetization-prepared rapid acquisition gradient-echo (MPRAGE) anatomical images were obtained using a gradient-echo sequence (TR = 1,900 ms, TE = 2.37 ms, TI = 900 ms, FOV = 250 mm, matrix = 288 × 288, slices per slab = 208, spatial resolution = 0.87 × 0.87 × 0.87 mm^3^). MRI scans in TN patients were acquired within the month preceding surgery.

### Pain Assessment

TN patients also completed a pain questionnaire prior to MRI acquisition in which they reported the side and distribution of their TN pain, and scored the severity of their pain attacks over the past week using a Visual Analog Scale (VAS, 0–100 mm).

### Texture Analysis

Image processing and subsequent voxel-wise analyses were conducted in Statistical Parametric Mapping 12 (SPM12; http://www.fil.ion.ucl.ac.uk/spm/software/spm12/) and Computational Anatomy Toolbox 12.1 (CAT12; http://dbm.neuro.uni-jena.de/cat12/). Images underwent bias correction and were segmented into gray and white matter in their native space. These native segments were used to create a whole-brain mask for each participant. T1-weighted images were normalized using the DARTEL (27) approach to the Montreal Neurological Institute (MNI) template provided by CAT12. The forward deformation fields were saved for later transformations into the standard space.

Texture analysis was performed using the gray level co-occurrence matrix (GLCM) method, a second-order statistical approach for extracting texture features (28). Details regarding the 3D adaptation and implementation of GLCM to generate 3D texture maps are provided elsewhere (19, 22). Here, whole-brain maps for three texture features—*autocorrelation, contrast*, and *energy*—were computed from T1-weighted images of all participants. Autocorrelation is defined as the measurement of the fineness and coarseness of texture and is related to the linear dependency of gray levels in a neighborhood of voxels. Contrast measures gray level variation, whereas energy quantifies the uniformity in the gray level distribution. The mathematical derivation of these texture features are provided elsewhere (19, 22, 29). The texture maps were normalized to the MNI template by applying the forward deformation fields obtained earlier and smoothed with a 6 mm FWHM Gaussian kernel for statistical analyses.

Three texture analyses were performed:

1. *Right-sided TN native-orientation analysis:* This analysis included only TN patients with right-sided pain (*n* = 9) and healthy control subjects. All images remained in their native orientation.
2. *All-TN native-orientation analysis:* This analysis included all TN patients (both right- and left-sided, *n* = 14) and healthy control subjects. All images remained in their native orientation.
3. *All-TN Ipsilateral orientation analysis:* This analysis included all TN patients (both right- and left-sided, *n* = 14). Correction for side-of-pain was carried out in the following manner: left-sided TN patients were left-right flipped such that the side-of-pain (ipsilateral) was now on the right side of the face. Right-sided TN patients were not flipped. Ipsilateral and contralateral texture features of TN patients were compared to healthy control subjects.

### Statistical Analysis

*Voxel-wise:* Statistical analysis of texture maps was restricted to four regions of interest determined *a priori* (ACC, brainstem, insula, thalamus) and defined according to the Harvard-Oxford Cortical Structural Atlas (30–33). A second-level full factorial model was designed to examine whole-brain between-group differences across the three texture features of interest while controlling for age. Group assignment was used as the factor of interest and age was regarded as a nuisance variable. Statistical significance was defined as *p* < 0.001 with a minimum cluster size of 20 voxels.

*Cluster-wise:* Region-of-difference masks were generated for each texture feature cluster found to differ between TN and healthy control subjects in the voxel-wise analysis. These masks were then applied to each subject (both TN patients and healthy control subjects), from which median raw texture values (absolute values) were calculated within each region-of-difference mask for each corresponding texture feature. Median raw texture values were then compared between TN and healthy control subjects using the Mann-Whitney *U*-test. Statistical significance was set at *p* < 0.006 after Bonferroni correction for multiple comparisons (*p* < 0.05/8 significant clusters = *p* < 0.006). These texture values represent absolute texture values in a region and were not normalized to a reference.

*Receiver-operator characteristic curve (ROC) analysis*: ROC analysis was performed using within region-of-difference mask median raw texture values to examine the ability of texture features to differentiate TN patients from healthy control subjects. Statistical significance was set at *p* < 0.05.

*Correlation of texture features with clinical variables:* In TN patients, Spearman correlation was performed between within region-of-difference mask median raw texture values and both: (1) duration of illness (in years); and (2) pre-operative pain score as measured using the VAS. Correlations were carried out individually for each of the three texture features. If, for a given texture feature, multiple clusters of difference between TN and healthy control subjects had been identified, raw texture values within the cluster showing the greatest area under the curve (AUC) with ROC analysis was used for correlation analysis. Statistical significance was set at *p* < 0.05.

## Results

### Patient Demographics

There were nine right-sided classical TN patients included in the *right-sided TN native-orientation analysis* (mean age: 57.1 ± 10.7, 4 F/5 M) with an average TN duration of 4.8 ± 3.2 years and mean preoperative pain VAS score of 85.3 ± 15.0. Five additional left-sided TN patients (mean age: 57.3 ± 15.8, 4 F/1 M, duration: 5.0 ± 3.1 years, pre-op VAS: 85.6 ± 14.9) were also included in the *all-TN native- and ipsilateral-orientation analyses* (*n* = 14, mean age 57.1 ± 12.1, 8 F/6 M). Average TN duration across these 14 patients was 4.7 ± 3.1 years, with mean preoperative pain VAS score 85.4 ± 14.4. Twenty healthy control subjects were included (age: 54.9 ± 9.4, 11 F/9 M) and used in all analyses. There were no differences between the groups of nine right-sided TN patients, 14 total TN patients, or healthy control subjects across any clinical or demographic variables (Table 1).

Table 1Demographic and clinical characteristics of ALL TN patients and healthy controls included in texture analysis.

**Table:** 

				**TN**		**HC**	***P*-value (2-tailed)**
**Number of subjects**				**14**		**20**	**N/A**
**Sex (Female/Male)**				**8/6**		**11/9**	**0.9014**
**Age (years)**				**57.14** **±** **12.13**		**54.88 ± 9.37**	**0.5442**
**Duration of TN (years)**				**4.86** **±** **3.08**		**N/A**	**N/A**
**Pre-operative VAS score (mm)**				**85.4** **±** **14.4**		**N/A**	**N/A**

**Table:** 

**TN patient**	**Sex**	**Age**	**Side**	**Duration (years)**	**Pre-op VAS (mm)**	**Affected trigeminal branch(es)**	**Medications**
1	M	57.5	R	8	65	V1	oxcarbazepine, baclofen
2	F	58.5	R	10	100	V2/3	carbamazepine
3	M	63.9	R	7	58	V1/2/3	carbamazepine
4	F	65.9	R	1	93	V2/3	carbamazepine, oxcarbazepine
5	F	64.9	R	7	100	V2/3	carbamazepine, pregabalin
6	M	41.8	R	2	89	V1/2	carbamazepine, pregabalin, baclofen, amitriptyline
7	F	36.3	R	3	89	V1/2	carbamazepine, gabapentin, lamotrigine
8	M	61.5	R	2.5	79	V3	carbamazepine
9	M	63.3	R	2.5	95	V2	oxcarbazepine, lamotrigine, baclofen
10	F	37.3	L	5	63	V1/2	carbamazepine, baclofen
11	M	45.1	L	9	98	V1	carbamazepine, pregabalin
12	F	75.1	L	3	81	V2	oxcarbazepine, pregabalin
13	F	60.4	L	7	86	V2/3	carbamazepine
14	F	68.5	L	1	100	V3	carbamazepine

*Student t-test and Chi-square test used where appropriate. VAS, visual analog pain scale. Means ± standard deviation are presented*.

### Between-Group Texture Differences

**Figure 1:**
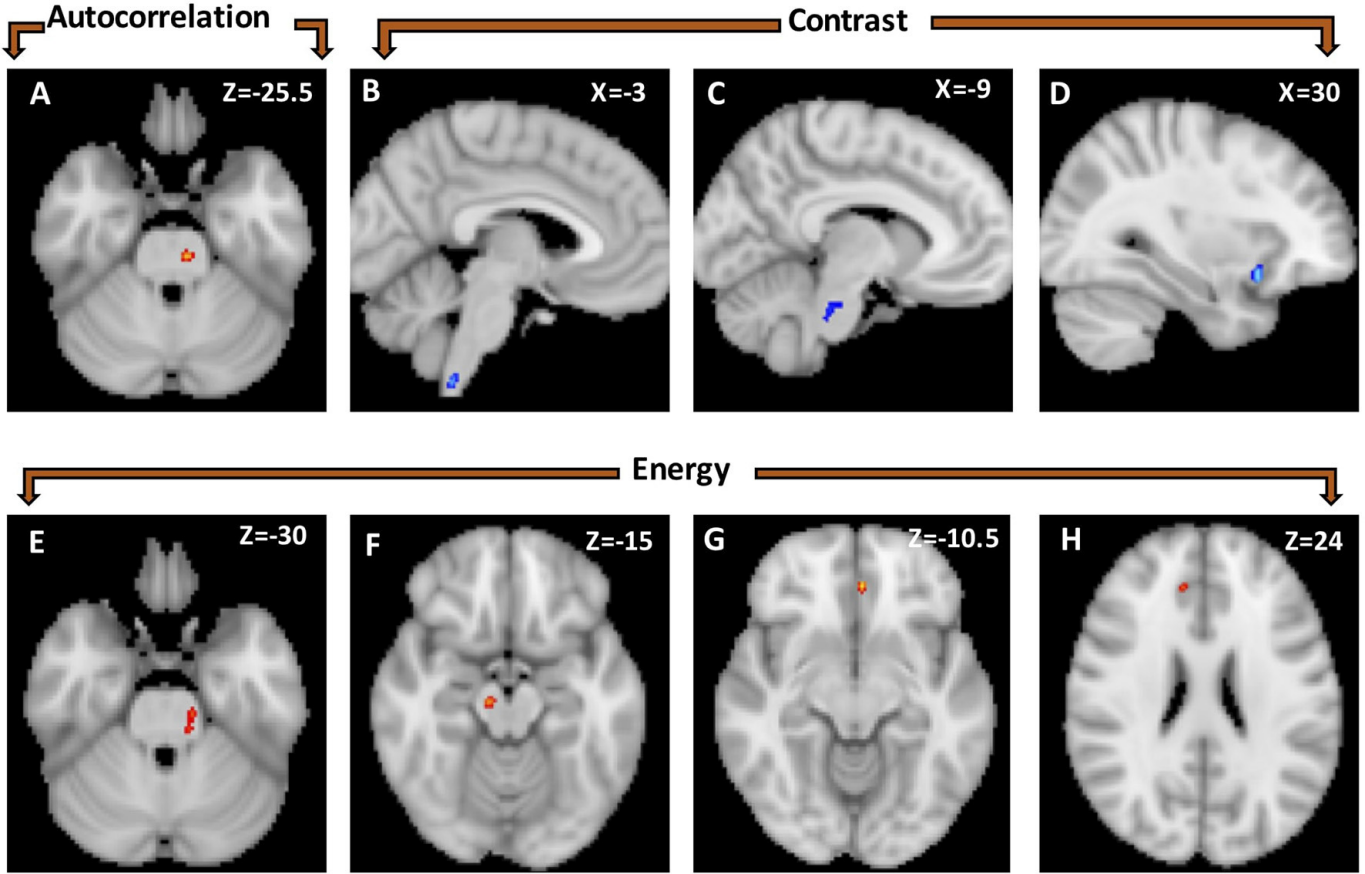
Statistical parametric map showing voxel-wise differences in texture features between healthy controls (*n* = 20) and right-sided TN patients (*n* = 9). Autocorrelation was greater in healthy controls than TN patients in a left brainstem cluster **(A)**. Contrast was greater in TN patients than healthy controls in three clusters: lower brainstem/cervical spinal cord **(B)**, left brainstem at the level of the trigeminal nerve **(C)** and right anterior insula **(D)**. Energy is greater in healthy controls than TN patients in four clusters located in the left brainstem **(E)**, right mid-brain **(F)**, and left **(G)** and right anterior cingulate **(H)**.

**Figure 2:**
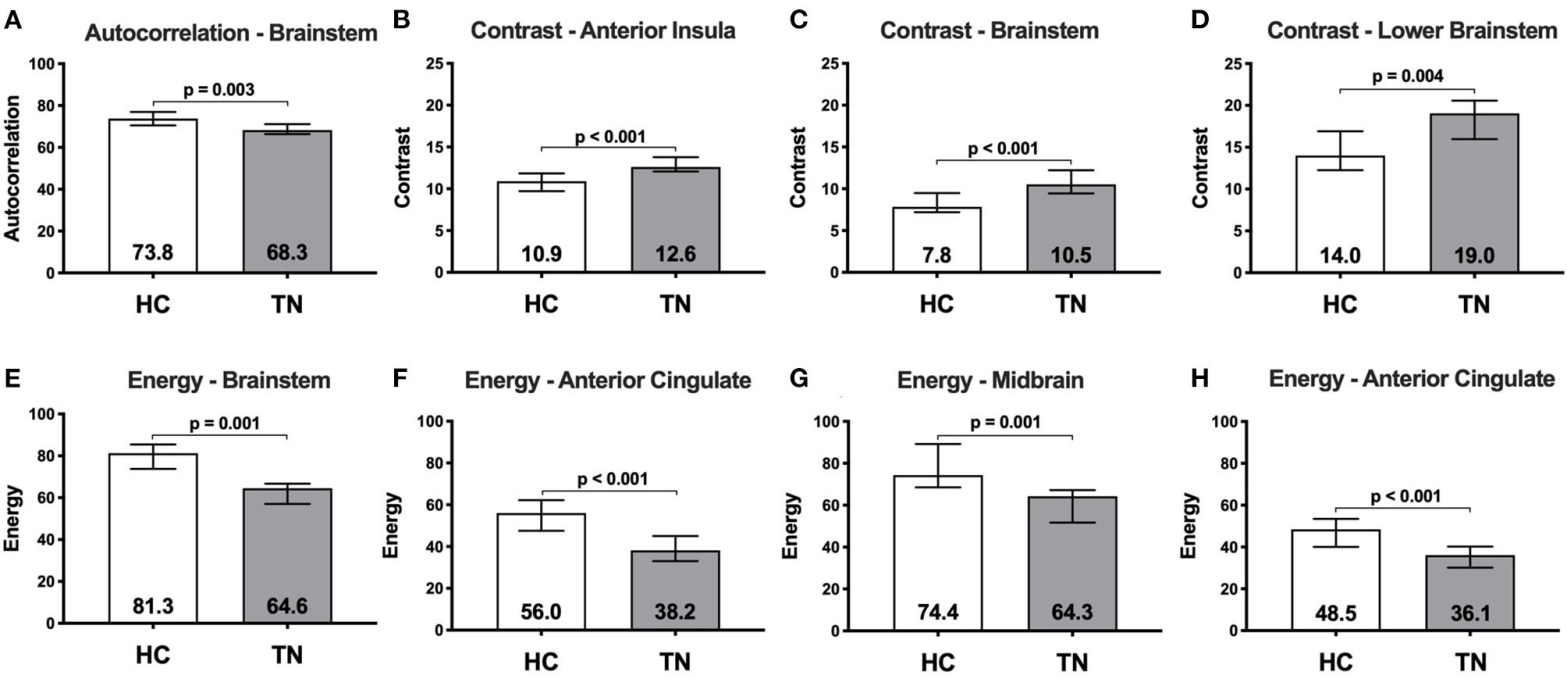
Median raw texture values within region-of-difference clusters between healthy controls (*n* = 20) and right-sided TN patients (*n* = 9). Autocorrelation was greater in healthy controls than TN patients in a left brainstem cluster **(A)**. Contrast is greater in TN patients than healthy controls in three clusters located in the: right anterior insula **(B)**; left brainstem at the level of the trigeminal nerve **(C)**; and lower left brainstem/spinal cord **(D)**. Energy is greater in healthy controls than TN patients in four clusters located in the: left brainstem **(E)**; left anterior cingulate **(F)**; right midbrain **(G)**; and right anterior cingulate **(H)**. Numbers within bars represent median, error bars represent interquartile range.

*Right-sided TN native-orientation analysis*: Autocorrelation is reduced in right-sided TN patients compared to healthy control subjects in the left brainstem at the level of the pons, just below the take-off of CNV, in both voxel- and cluster-wise analyses (Table 2, Figures 1A, 2A). Contrast is increased in right-sided TN patients compared to healthy control subjects in the left brainstem and right anterior insula in both voxel- and cluster-wise analyses (Table 2, Figures 1B–D, 2B–D). Energy is reduced in right-sided TN patients compared to healthy control subjects in the left and right ACC, right midbrain, and left brainstem in both voxel- and cluster-wise analyses (Table 2, Figures 1E–H, 2E–H). We did not observe any differences in texture features within the thalamus.

**Table 2:** Statistical parametric map and region of interest (ROI) analysis results between right-sided trigeminal neuralgia patients (TN) and healthy controls (HC).

**Texture feature Region**	**MNI**			***t*-stat (peak)**	**Cluster size**	**Region-of-difference raw texture value**		***P*-value** ** (2-tailed)**
	**X**	**Y**	**Z**			**TN**	**HC**	
**Autocorrelation**								
Brainstem (left)	−6	−25.5	−25.5	4.27	56	68.3 (66.4, 71.2)	73.8 (70.5, 77.0)	0.003
**Contrast**								
Anterior insula (right)	30	16.5	−13.5	4.93	41	12.6 (12.1, 13.8)	10.9 (9.7, 11.8)	<0.0001
Lower brainstem/cervical	−3	−45	−63	4.55	35	19.0 (16.0, 20.6)	14.0 (12.3, 16.9)	0.0043
spinal cord (left)								
Brainstem (left)	−9	−30	−30	3.77	46	10.5 (9.5, 12.2)	7.8 (7.2, 9.5)	0.0004
**Energy**								
Anterior cingulate (left)	−3	39	−10.5	4.31	54	38.2 (33.1, 45.0)	56.0 (47.6, 62.2)	0.0001
Midbrain (right)	10.5	−19.5	−15	4.07	37	64.3 (51.7, 67.2)	74.4 (68.6, 89.2)	0.0011
Anterior cingulate (right)	10.5	37.5	24	3.94	35	36.1 (30.1, 40.2)	48.5 (40.0, 53.5)	0.0020
Brainstem (left)	−12	−22.5	−30	3.88	79	64.6 (57.0, 66.7)	81.3 (73.8, 85.5)	0.0011

*Region, significant cluster regions; MNI, Montreal Neurologic Institute coordinates; t-stat (peak), peak t-stat within cluster; region-of-difference raw texture value, median raw texture values within regions of difference (range of raw texture values indicated in parentheses); p-value (2-tailed), Mann-Whitney U test comparing median raw texture values between TN and HC within regions of difference*.

*All-TN native- and ipsilateral-orientation analyses:* There are no differences between all TN patients and healthy control subjects within any ROIs for autocorrelation, contrast, or energy in either native- or ipsilateral orientation.

### Receiver-Operator Characteristic Curve Analysis

**Figure 3:**
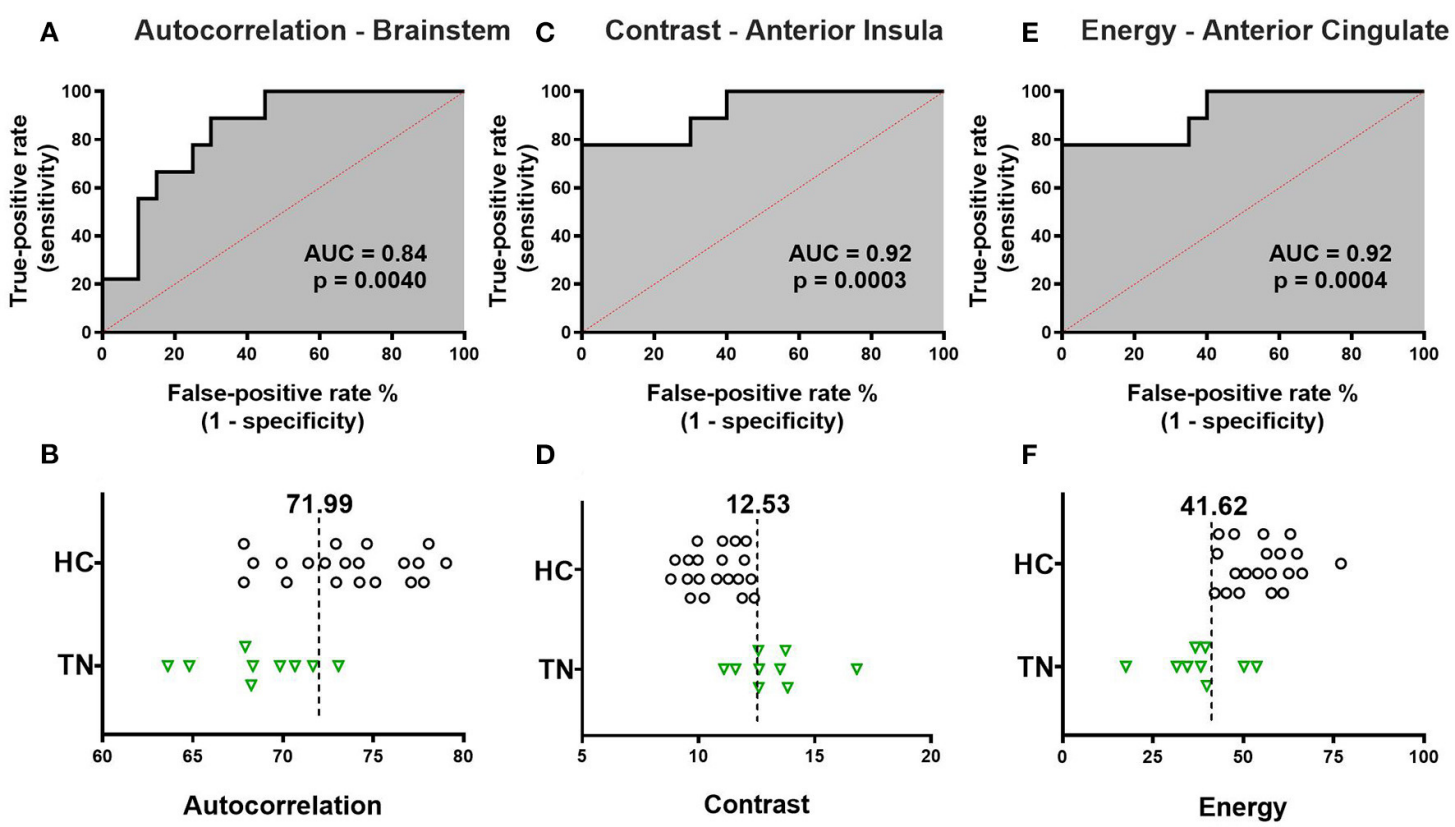
Receiver-operator characteristic (ROC) curve analysis of texture features. The single ROC curve with the largest area under the curve (AUC) is displayed for each texture feature. **(A)** ROC curve for left brainstem autocorrelation has AUC 0.839 (*p* = 0.0040). The optimal operating threshold autocorrelation value of 71.99 is 89% sensitive and 70% specific for subject group. **(B)** Left brainstem autocorrelation values for each individual subject are displayed with optimal operating threshold autocorrelation value overlaid. **(C)** ROC curve for right anterior insula contrast has AUC 0.922 (*p* = 0.0003). The optimal operating threshold contrast value of 12.53 is 78% sensitive and 100% specific for subject group. **(D)** Right anterior insula contrast values for each individual subject are displayed with optimal operating threshold contrast value overlaid. **(E)** ROC curve for left ACC energy and subject group has AUC 0.917 (*p* = 0.0004). The optimal operating threshold energy value of 41.62 is 78% sensitive and 100% specific for subject group. **(F)** Left ACC energy values for each individual subject are displayed with optimal operating threshold energy value overlaid. TN, right trigeminal neuralgia patients; HC, healthy control subjects.

ROC analysis was performed for each texture feature and corresponding region-of-difference cluster displayed in Table 2 in right-sided TN patients. ROC curves for all eight region-of-difference clusters and corresponding texture features successfully segregate TN patients from healthy control subjects. Figure 3 shows the single ROC curve with the greatest AUC for each texture feature (Figure 3). The ROC curve for left brainstem autocorrelation cluster (Figure 1A) has an AUC of 0.839 (*p* = 0.004, Figure 3A), and is 89% sensitive and 70% specific for subject group at an optimal operating threshold autocorrelation value of 71.99 (Figure 3B). The ROC curve for the right anterior insula contrast cluster (Figure 1D) has an AUC of 0.922 (*p* = 0.0002, Figure 3C), and is 78% sensitive and 100% specific for subject group at an optimal operating threshold contrast value of 12.53 (Figure 3D). The ROC curve for the left ACC energy cluster (Figure 1G) has an AUC of 0.917 (*p* = 0.0004, Figure 3E), and is 78% sensitive and 100% specific for subject group at an optimal operating threshold energy value of 41.62 (Figure 3F).

### Correlation Between Texture and Clinical Features

**Figure 4:**
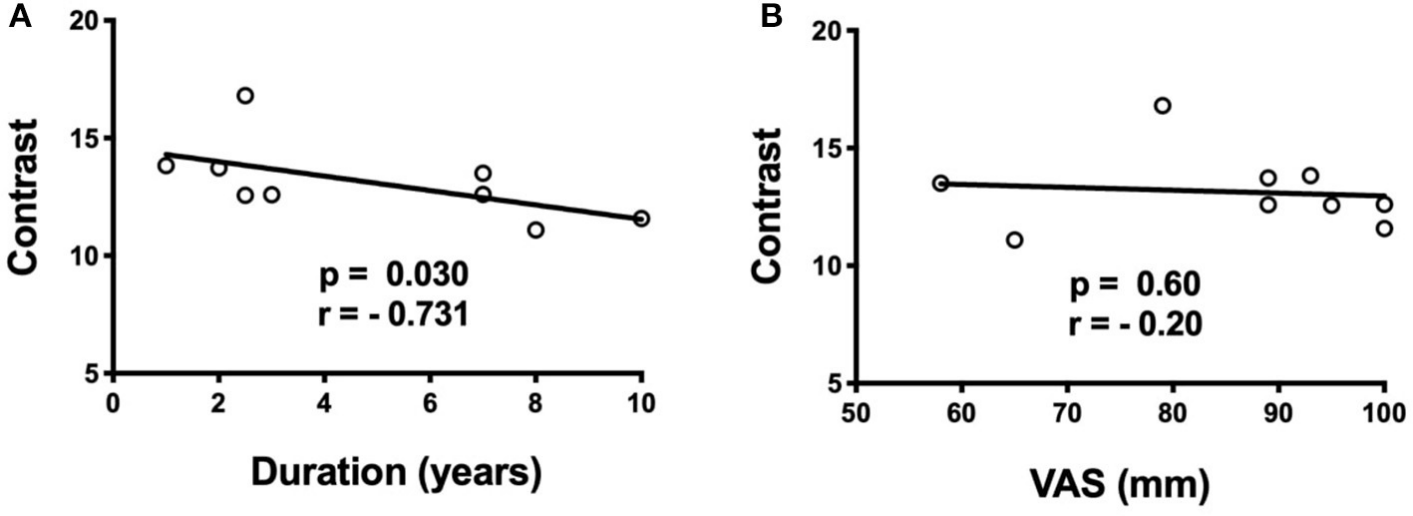
Spearman correlations of anterior insula cluster contrast with duration of TN (years) and pre-operative pain score (VAS). **(A)** There is a negative correlation (*p* = 0.030, *r* = −0.73) between contrast in the right anterior insula and duration of TN, **(B)** but no correlation with pre-operative VAS score.

There is a significant negative correlation between average raw contrast in the right anterior insula region-of-difference cluster and duration of TN (*p* = 0.030, and *r* = −0.73), but there is no correlation between average raw contrast in the right anterior insula and pre-operative VAS pain score (Figures 4A,B). There are no significant correlations between duration of TN or pre-operative pain score with either left brainstem autocorrelation or left ACC energy (Supplementary Figure 1).

## Discussion

In this study, we used texture analysis to identify differences in brain structure between classical TN patients and healthy subjects. To our knowledge, this is the first application of texture analysis to a neuroimaging study of TN or chronic pain. Our hypothesis-driven design confirmed that texture abnormalities overlap spatially with previous-reported structural abnormalities in the TN brain, specifically in the ACC and anterior insular regions. Texture abnormalities were also found in the brainstem in TN; while long suspected, brainstem structural abnormalities of this kind have rarely been identified in TN using other neuroimaging approaches. Not only do these texture abnormalities distinguish TN from healthy control subjects, but in some cases, they also correlate with clinical features (i.e., with duration of illness in the case of the anterior insular region). Taken together, these findings yield further evidence that TN is not merely characterized by structural alterations of CNV, but rather involves key structural brain changes as do other chronic pain conditions. Further, the findings suggest that the application of texture analysis to investigate TN serves not only as a useful confirmation of previous structural brain imaging studies but may also yield further insights into the pathoanatomy of the disease.

Using texture analysis, we found evidence of brainstem abnormalities in right-sided TN patients. These findings are in line with reports of brainstem diffusivity differences—measured using DTI—between TN patients and healthy subjects (11–13). Of note, alterations in brainstem diffusivity have primarily been observed immediately proximal to the CNV root entry zone on the affected side. By contrast, we observed brainstem texture abnormalities contralateral to the side of pain, extending as far caudally as the lower medulla/upper cervical spinal cord. We speculate that these abnormalities may reflect abnormalities of 2nd order trigeminal afferents ascending in the trigemino-thalamic tract after their medullary decussation. We and others have previously identified volume change in the thalamus of patients with TN, suggesting that trigeminal system abnormalities may extend up to 3^rd^ order afferent neurons residing in the ventro-posteromedial nucleus of the contralateral thalamus (15, 17). Consequently, we were surprised not to find texture abnormalities in the thalamus as well, though, this may be related to our small sample size, or else it may simply be the case that texture analysis and other structural imaging analysis approaches—such as gray-matter volumetry or voxel-based morphometry—have different sensitivities to detect abnormalities within this particular brain region.

Limbic system involvement in chronic pain is widely reported (34–37), and neuroimaging studies of TN and other chronic pain conditions repeatedly identify structural abnormalities across several limbic regions (14, 15, 17, 38–40). In particular, structural alteration of the ACC is one of the most consistently observed brain abnormalities across pain conditions using various imaging modalities (15, 34, 35, 39). In agreement with these previous findings, we also identified texture abnormalities in this important pain-relevant structure. Given the ACC's critical role in determining the *unpleasantness* of pain (34, 41), we had predicted that ACC texture abnormalities might correlate with pre-operative pain score, though this was not the case. One explanation of this is that VAS may provide an inaccurate picture of pain severity in classical TN, since patients with the disorder have intermittent pain attacks interspersed with pain-free periods, and as such may not be able to characterize severity with a single numerical rating (42). Furthermore, given the episodic nature of TN, it may be the case that structural abnormalities in brain regions involved in the *perception of pain*—as opposed to those involved in pain unpleasantness—may be more likely to correlate with clinical features.

Indeed, a critically important structure in pain perception is the insula, which is a key node in the process of determining salience. Which sensory information is brought forward to conscious awareness is largely the responsibility of the anterior insular region, and, more specifically, raising awareness of painful stimuli appears to be a function of the right anterior insula (36). Our detection of abnormal texture in the right anterior insula aligns with this structure's role in salience and pain perception. Moreover, pre-operative right anterior insular cortical thinning has been described in patients with TN and chronic osteoarthritis of the hip, and has also been shown to normalize following surgical treatment in both conditions (14, 43). Furthermore, we observed a significant correlation between right anterior insula texture and duration of TN, suggesting that structural change to the insula may result from the long-term experience of chronic TN pain, or perhaps that this structure is involved in central sensitization. It remains to be determined whether this kind of structural alteration of the insula is unique to TN, or is more generally associated with treatment resistance in chronic pain conditions. A full evaluation of long-term surgical outcome of patients enrolled in the current data set, and the relationship of outcome to both pre-operative texture abnormalities and texture changes following surgery, is currently being undertaken.

An interesting observation in this study is that all significant texture-related findings arose from the *right-sided native-orientation* analysis despite *all-TN native-orientation* and *all-TN ipsilateral-orientation* analyses having a larger sample size. These findings suggest that texture abnormalities in TN reflect the combined influence of both the absolute side (i.e., left, right) and relative side (i.e., ipsilateral, contralateral) of pain. It is well-known that TN displays a right-sided propensity, with a right-sided TN:left-sided TN ratio approaching 1.5:1 in classical TN (9, 10, 44, 45). Additionally, hemispheric lateralization of function has been observed for both the ACC and insula, with the right-side in each case being more clearly linked to chronic pain (34, 36). Our results suggest that brain imaging studies in TN need to take into account the potential effect of side-of-pain when interpreting structural neuroimaging data. They also suggest that there may be brain-based contributions to the right-sided predilection of TN, notwithstanding possible asymmetries of the trigeminal nerve itself or the cranial foramina through which its branches traverse (10), the existence of which, to date, have not been supported by strong evidence.

Texture analysis is a relatively simple-to-implement, semi-automated technique applied to T1-weighted images. This approach may be particularly attractive to clinical researchers in that it is applicable to T1-weighted MR images which are the mainstay of standard clinical imaging in TN; consequently, retrospective evaluation of large clinically acquired T1-weighted MRI datasets with texture analysis is highly feasible. Additionally, unlike other automated techniques, texture analysis also provides voxel-level raw texture values, allowing for simple individual-level scalar comparisons conducive to clinical application (diagnostic or prognostic). While the biological significance of texture and textural changes remain an area of active investigation, texture is likely an amalgamation of large- and small-scale physiological influences. Texture findings have been shown to overlap spatially with observations from other imaging modalities—as we found in the present study— suggesting that while texture abnormalities are not yet fully understood, they are likely to be clinically or pathophysiologically relevant (22, 24). For example, well-established structural imaging signatures of brain pathology have been reproduced using texture analysis in Alzheimer's disease (19, 46), and recent bodies of work from multiple sclerosis and amyotrophic lateral sclerosis have identified relationships between histological findings (i.e., gliosis and neuronal loss) and texture (47, 48). Together, these findings suggest that features of texture are biologically relevant and may be clinically useful. Admittedly, more work is needed to further understand this relationship in trigeminal neuralgia.

This study is not without limitations. As mentioned above, the sample size of 14 TN patients (nine right-sided TN patients) and 20 healthy subjects, is relatively small and potentially underpowered. Additionally, sample size asymmetry (i.e., 9 RTN vs. 20 healthy controls) may have further limited our sensitivity to detect differences between groups that would have otherwise surpassed the threshold for statistical significance if the groups were more balanced (49). Therefore, the findings of this study must be validated, and expanded upon, in future larger sample investigations. Having said that, texture analysis—unlike other automated volumetric approaches such as voxel-based morphometry—generates *raw* scalar value outputs, allowing for simple, univariate statistical evaluations of between-group differences conducive to smaller sample studies. We found that these group-level differences in raw texture values were large—approximately 20% when averaged across all inter-group comparisons—further increasing confidence that the texture abnormalities we observed are real. Next, the potential influence of MRI acquisition parameters and scanner manufacturer on texture features is another potential critique (22); the results reported here will need to be replicated in patients scanned using different scanners, ideally in a multi-center approach. Another potential methodological limitation is that texture analysis may be confounded by common aging-related conditions such as hypertension and diabetes, which, as a result of small vessel disease, may cause subtle alterations of brain structure—and therefore texture features—across several regions. Perhaps the most important limitation is that our data cannot answer the question as to whether brain texture abnormalities are a cause or a consequence of TN, since we analyzed only a single time point during the trajectory of the illness. The correlation we observed between right anterior insula texture and duration of illness suggests that certain texture changes evolve over the course of the disease as it becomes increasingly medically-refractory, though the relatively short average duration of TN across patients in this study (i.e., <5 years) may argue against this. As mentioned above, a focus of future work will be longitudinal analysis of texture changes in the brain over the longitudinal course of TN, including a comparison of pre-surgical and post-surgical timepoints.

In summary, our novel application of texture analysis to T1-weighted MRI scans in patients with classical TN shows that there are significant texture abnormalities in several pain-relevant brain regions that can segregate TN patients from unaffected healthy controls. These findings further support the notion that nerve-centric conceptualizations of TN are, on their own, incomplete. Establishing the clinical relevance of texture analysis in TN and other chronic pain conditions is a worthwhile area for further investigation.

## Data Availability Statement

The raw data supporting the conclusions of this article will be made available by the authors, without undue reservation.

## Ethics Statement

The studies involving human participants were reviewed and approved by the REB 3: Health Research Ethics Board - Health Panel of the University of Alberta. The patients/participants provided their written informed consent to participate in this study.

## Author Contributions

HD designed the project, was responsible for all patient data collection, contributed to ethics approval, performed data analysis and results interpretation, and wrote the manuscript. AI contributed to data analysis, results interpretation, and manuscript generation. DT contributed to data analysis and manuscript generation. YY developed the texture analysis toolbox utilized in the study and manuscript generation. BW contributed to study design and patient recruitment. SK developed the texture toolbox utilized in the study, results interpretation, and manuscript generation. TS oversaw all aspects of the study, and directly contributed to study design, ethics approval, data analysis, results interpretation, and manuscript generation. All authors contributed to the article and approved the submitted version.

## Conflict of Interest

The authors declare that the research was conducted in the absence of any commercial or financial relationships that could be construed as a potential conflict of interest.

## Abbreviations

TN: trigeminal neuralgia
CNV: cranial nerve V
MRI: magnetic resonance imaging
DTI: diffusion tensor imaging
ACC: anterior cingulate cortex
MPRAGE: 3D magnetization-prepared rapid acquisition gradient-echo
ROC: receiver-operator characteristic curve
VAS: visual analog scale
AUC: area under curve.
