# Supplementary material for: MRI Texture Analysis Reveals Brain Abnormalities in Medically Refractory Trigeminal Neuralgia
Source: Front Neurol. 2021 Feb 12;12:626504. doi: 10.3389/fneur.2021.626504 (PMC7907508; doi:10.3389/fneur.2021.626504)
Supplement: Supplementary file 1 [file Image_1.pdf]

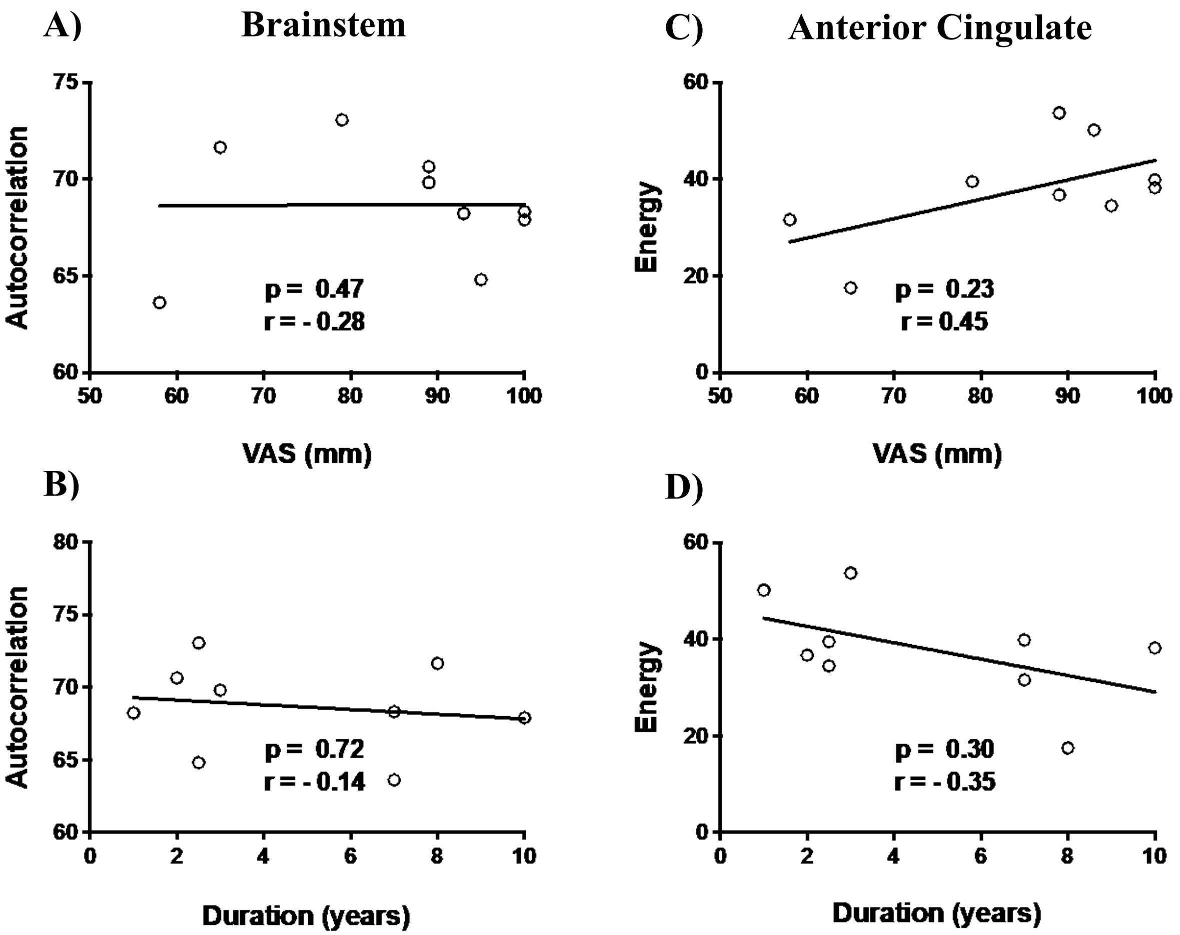

Supplementary Figure 1: Spearman correlations of cluster texture feature values with pre-operative pain score (VAS) and duration of TN (years). There are no significant correlations between autocorrelation in the left brainstem and pre-operative pain score (**A**) or duration of TN (**B**), or between energy in the left anterior cingulate cortex (ACC) and pre-operative pain score (**C**) or duration of TN (**D**).
